# Automatic Lifestate Identification and Clustering

**DOI:** 10.23889/ijpds.v8i3.2274

**Published:** 2023-09-11

**Authors:** Sam Smith, Gavin Smith, John Harvey

**Affiliations:** 1 N/LAB, University of Nottingham

## Introduction & Background

Summarising high-dimensional time series data across multiple entities is an increasingly prevalent problem because mass data collection has become routine in most domains. We propose a method of automatically summarising high-dimensional data.

## Objectives & Approach

Summarization in such a context is both with regard to a reduction of the high-dimensional observations and large number of temporal points. While numerous methods to segment and/or summarise time series exist, the properties often do not align with the needs of consumers of the summaries or require the unrealistic setting of parameters. Addressing this, we define a set of broad properties that lead to high utility in a broad class of domains, which are determined by an information theoretic notion of optimality. Intuitively these properties reflect the summarization of such data into lifestates where (1) the number of possible lifestates is limited and shared across entities to allow interpretation and comparison and (2) the number of lifestate-transitions is jointly controlled to provide a parameterless, optimal summarization of both the high sample and temporal dimensionality.

## Relevance to Digital Footprints

Example data include: regular survey collection, consumer purchasing history from transactional data (where the number of possible items to choose from is high), or other repeatedly sampled digital data. Within the Digital Footprints domain, concise descriptions of high-dimensional data (summarizations) are extremely important. For example, lifestates within health records could be identified and used to find critical patterns in the decline or recovery of patients.

## Conclusions & Implications

This work aims to find segmentations that optimally trade off the number of states and segments that humans must then interpret, while still capturing salient state changes. Building on prior work, we propose a model with complexity controlled by normalised maximum likelihood (NML). In short, the proposed model generates automated summarizations that are both optimally concise and informationally rich, according to information theory, a branch of mathematics.

